# A Phase I/II study of vesigenurtacel-l (hs-410) or placebo in combination with Bacillus Calmette-Guérin (BCG) in patients with non-muscle invasive bladder cancer (NMIBC)

**DOI:** 10.1186/2051-1426-2-S3-P81

**Published:** 2014-11-06

**Authors:** Gary D Steinberg, Neal Shore, Lawrence Karsh, James Bailen, Michael Woods, Mark Schoenberg, Taylor H Schreiber, Melissa Price

**Affiliations:** 1 University of Chicago Medical Center, Chicago, IL, USA; 2 Carolina Urologic Research Center, Myrtle Beach, SC, USA; 3 The Urology Center of Colorado, Denver, CO, USA; 4 First Urology, USA; 5 The University of North Carolina at Chapel Hill, Chapel Hill, NC, USA; 6 Montefiore Medical Center, New York, NY, USA; 7 Heat Biologics, Durham, NC, USA

## Background

Vesigenurtacel-L is an allogeneic cell-based therapeutic cancer vaccine that was selected based on its expression of a range of tumor antigens (surviving, LAGE-1, MAGE-A3, MAGE-A11, PRAME and others) that are known to be expressed amongst a high proportion of primary bladder tumors. The parental cell line was transfected with an engineered secretable heat shock protein, gp96-Ig, which functions dually as an antigen delivery vehicle and adjuvant. Importantly, our immune systems evolved to screen for extracellular gp96 as a defense mechanism against intracellular pathogens, and this mechanism of immune activation preferentially stimulates activation of CD8+ cytotoxic T cells through the antigen cross-presentation pathway. Thus, combination therapy with Vesigenurtacel-L is proposed to activate patient CD8+ T cell responses against a variety of bladder tumor antigens, which may provide additional benefit in combination with a local inflammatory therapy such as BCG to prevent recurrence and eliminate minimal residual disease following initial surgery.

## Methods

This is a Phase I/II study of 84 patients with non-muscle invasive bladder cancer who have undergone TURBT, are candidates for BCG treatment, are judged to be at an increased risk for recurrence, and are either BCG naïve or have completed previous BCG treatment >12 months prior to the most recent TURBT. Nine patients will be treated in the Phase I portion with monotherapy Vesigenurtacel-L (10^6 ^cells per dose, 12 weekly doses followed by 3 monthly doses) after induction BCG. In the Phase II portion, 75 patients will be randomized 1:1:1 to one of two doses of Vesigenurtacel-L (either 10^6 ^or 10^7 ^cells/dose) or placebo in combination with 6 weeks of induction BCG, followed by 6 weeks of monotherapy Vesigenurtacel-L in the induction Phase. Maintenance treatment in combination with BCG will continue in patients without evidence of disease for 3 courses of 3-weekly treatments at the following time points relative to the initial TURBT: 3 months, 6 months, and 12 months. The primary endpoint in Phase I is safety; the Phase II primary endpoint is 1-year recurrence-free survival. Secondary efficacy evaluations include determination of recurrence and progression at various time points, analyses of immunologic response in peripheral blood, and antigen expression analysis in tissue. Clinical trial information: NCT02010203.

